# Human Amnion Epithelial Cells: A Potential Cell Source for Pulp Regeneration?

**DOI:** 10.3390/ijms23052830

**Published:** 2022-03-04

**Authors:** Cristina Bucchi, Ella Ohlsson, Josep Maria de Anta, Melanie Woelflick, Kerstin Galler, María Cristina Manzanares-Cespedes, Matthias Widbiller

**Affiliations:** 1 Research Centre for Dental Sciences (CICO), Department of Integral Adult Dentistry, Faculty of Dentistry, Universidad de La Frontera, Temuco 4811230, Chile; 2 Department of Conservative Dentistry and Periodontology, University Hospital Regensburg, 93053 Regensburg, Germany; ella.ohlsson@ukr.de (E.O.); melanie.woelflick@ukr.de (M.W.); matthias.widbiller@ukr.de (M.W.); 3 Human Anatomy and Embryology Unit, Department of Pathology and Experimental Therapeutics, Faculty of Medicine and Health Sciences, Campus de Bellvitge, Universitat de Barcelona, 08907 L’Hospitalet de Llobregat, Spain; janta@ub.edu (J.M.d.A.); mcmanzanares@ub.edu (M.C.M.-C.); 4 Department of Conservative Dentistry and Periodontology, Friedrich-Alexander-University Erlangen-Nürnberg, 91054 Erlangen, Germany; kerstin.galler@uk-erlangen.de

**Keywords:** human amnion epithelial cells, dental pulp stem cells, dentin matrix proteins, odontoblastic differentiation, revitalization

## Abstract

The aim of this study was to analyze the suitability of pluripotent stem cells derived from the amnion (hAECs) as a potential cell source for revitalization in vitro. hAECs were isolated from human placentas, and dental pulp stem cells (hDPSCs) and dentin matrix proteins (eDMPs) were obtained from human teeth. Both hAECs and hDPSCs were cultured with 10% FBS, eDMPs and an osteogenic differentiation medium (StemPro). Viability was assessed by MTT and cell adherence to dentin was evaluated by scanning electron microscopy. Furthermore, the expression of mineralization-, odontogenic differentiation- and epithelial–mesenchymal transition-associated genes was analyzed by quantitative real-time PCR, and mineralization was evaluated through Alizarin Red staining. The viability of hAECs was significantly lower compared with hDPSCs in all groups and at all time points. Both hAECs and hDPSCs adhered to dentin and were homogeneously distributed. The regulation of odontoblast differentiation- and mineralization-associated genes showed the lack of transition of hAECs into an odontoblastic phenotype; however, genes associated with epithelial–mesenchymal transition were significantly upregulated in hAECs. hAECs showed small amounts of calcium deposition after osteogenic differentiation with StemPro. Pluripotent hAECs adhere on dentin and possess the capacity to mineralize. However, they presented an unfavorable proliferation behavior and failed to undergo odontoblastic transition.

## 1. Introduction

Regenerative endodontics refers to biologically based treatment procedures, e.g., revitalization, for immature necrotic teeth [1]. It aims at the restoration of the pulp’s physiology, including its immune, sensory and secretory functions, to improve the long-term prognosis of the tooth [2]. Over the last two decades, in vivo studies have shown satisfactory clinical outcomes with healing of periapical lesions [3] and resolution of clinical symptoms [4,5], as well as root thickening and lengthening [4] or apical closure [6]. However, the newly formed hard tissue does not resemble dentin but an ectopic tissue similar to cementum [7] or osteodentin [8], while the soft tissue lacks the pulp’s characteristic organization and cells with a distinct odontoblast phenotype [9]. The absence of odontoblasts after revitalization and the formation of a tissue other than dentin might compromise both the capability of the treated teeth to react to future injuries and also their biomechanical performance [10].

Novel and more elaborate approaches are being tested to overcome the lack of regeneration after the classical approach of revitalization and to achieve more predictable histological outcomes [11]. In this context, tissue engineering relies on the delivery of stem cells and/or recombinant growth factors in a scaffold into the root canal to facilitate pulp regeneration. These methods can be subcategorized into the cell homing approach, which utilizes signaling molecules to induce the migration, proliferation and differentiation of stem cells from the periapical tissues [12,13], and the cell transplantation approach [14]. The latter relies on the delivery of stem cells able to form new pulp tissue in the root canal [15].

To be considered as regenerated dental pulp, the newly formed tissue in the root canal must be vascularized as well as innervated, contain a similar cell density and microarchitecture to natural pulp and give rise to new odontoblast cells located at the dentin–pulp interface that are able to secrete tubular dentin in the course of tooth development but also at later time points [16]. In the context of pulp regeneration, the re-establishment of an odontoblast layer seems to be crucial due to its central role in tooth physiology and pathology [17,18,19]. Located at the dentin–pulp interface, these cells are the first line of defense against a bacterial invasion [20], they release antimicrobial agents [21] and have an immunomodulatory potential [22], allowing the tooth to immediately respond to stimuli, e.g., by secretion of dentin [17]. They also possess sensory functions by transducing pH changes and pressure as well as other pain-related stimuli [18]. Thus, it is of great interest for dental pulp tissue engineering to identify cell sources that are capable of differentiating into odontoblasts.

Recent in vivo studies have shown that pulp regeneration is possible after stem cell transplantation [23,24]. Histology revealed newly differentiated odontoblast-like mineralizing cells in contact with dentin. This approach is based on the transplantation of previously isolated and expanded autologous dental pulp stem cells (hDPSCs) and has proven successful in clinical trials [14,25]. In vitro studies also display odontogenic differentiation and the mineralization potential of hDPSCs when cultured with dentin matrix proteins (eDMPs) [26]. Dental pulp stem cells express dentin sialoprotein and differentiate into odontoblast-like cells with cellular processes extending into the dentinal tubules when seeded into EDTA-conditioned dentin cylinders and transplanted subcutaneously into immunocompromised mice [27]. However, the cell transplantation approach using hDPSCs and other tooth-derived cell types is challenging due to the necessity of cell expansion to obtain a sufficient number of cells, the need for a donor tooth and the limited differentiation potential of the multipotent stem cells compared to pluripotent stem cells [28].

A potential cell source to overcome those obstacles might be the amnion [29], the innermost layer of the human placenta. It contains amniotic epithelial cells (hAECs), which are formed by day 8 after fertilization and therefore maintain the plasticity of pre-gastrulation cells. Thus, hAECs are able to differentiate into cells of all three embryological layers [30], whereas multipotent stem cells, such as hDPSCs, are only capable of differentiating into cell types of one germ layer [31]. Human AECs showed the expression of human embryonic and pluripotent stem cell markers [30], such as stage-specific embryonic antigen-4 (*SSEA4)*, octamer-binding transcription factor 4 (*OCT4)* and nanog homebox (*NANOG)* [32]. Moreover, hAECs reportedly have antimicrobial properties [33], immunomodulating potential [34] and can induce angiogenesis [35], which makes a useful cell type for regenerative therapies [29]. Up to 300 million hAECs can be obtained from one human placenta by a simple isolation protocol [29] which may be either expanded, directly applied or cryopreserved, e.g., in cell banks [29], which would ease the provision of these pluripotent stem cells. They are already used to treat several medical conditions, e.g., liver diseases and Parkinson’s disease [36,37]. Moreover, amnion epithelial cells are not tumorigenic [32,38] and do not elicit an immune response upon heterologous transplantation, since they express very low levels of leukocyte antigens [30,32].

Although some exceptional studies have tested the transplantation of amnion membrane into the root canal with satisfactory clinical outcomes [39], no study has assessed the potential of hAECs to be used for endodontic regeneration. Thus, the aim of this study was to evaluate hAECs as a potential cell source for dental pulp tissue engineering. It was hypothesized that both hAECs and hDPSCs provide similar qualities in terms of viability, dentin adherence, odontoblast-like differentiation and mineralization.

## 2. Results

### 2.1. Isolation and Characterization of hAECs

**Figure 1:**
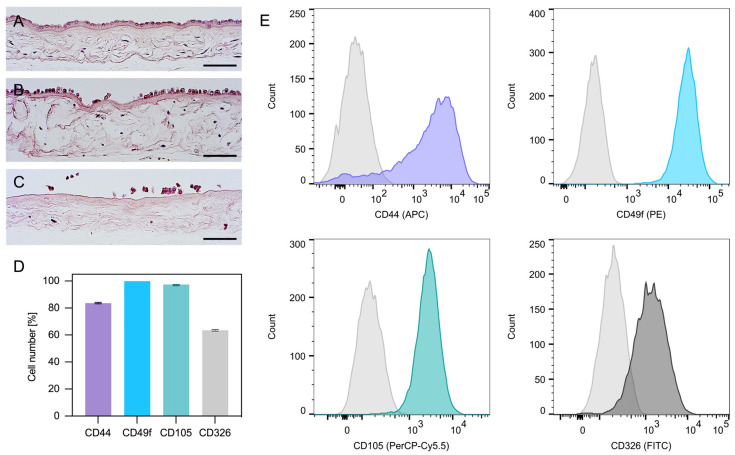
Amnion staining and expression profile of hAECs. Amnion before digestion (**A**) and after the first (**B**) and second digestion (**C**). The hAECs were attached to a collagen membrane forming a monolayer of columnar/cuboidal cells (hematoxylin and eosin; scale bars: 100 µm). Expression profile of hAECs determined by flow cytometry analysis (**D**). The hAECs in culture expressed both mesenchymal markers (CD44 and CD105) as well as epithelial markers (CD49f and CD326) (**D**,**E**).

As shown by the histological analysis, the first digestion of the amnion only partially detached hAECs (Figure 1A,B); however, the second digestion released nearly all cells (Figure 1C). Interestingly, the flow cytometric analysis of hAECs in culture revealed both epithelial (CD49f, CD326) and mesenchymal (CD105, CD44) surface antigens (Figure 1D,E).

### 2.2. Cell Viability

**Figure 2:**
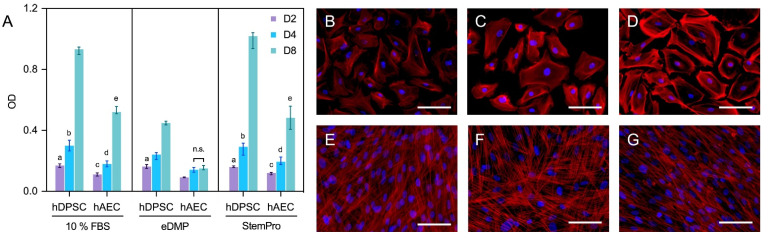
Viability and morphology of hAECs and hDPSCs. Cell viability of hAECs and hDPSCs cultured with eDMP and StemPro after 2, 4 and 8 days (**A**). Median values and 25–75% percentiles were calculated from three independent experiments performed in triplicate (*n* = 9). Fluorescence microscopy of hAECs and hDPSCs cultured with different media after 7 days and stained with DAPI and phalloidin (**B**–**G**). Cells were cultured in DMEM with 10% FBS (**B**,**E**), with eDMP (**C**,**F**) and with StemPro (**D**,**G**). hAECs exhibit a cobblestone-like morphology (**B**–**D**) while hDPSCs exhibit a mesenchymal stem cell phenotype (**E**–**G**). (Scale bars: 50 µm).

Human amnion epithelial cells showed a reduced viability compared to hDPSCs in all groups and at all time points (Figure 2A). Neither eDMPs nor StemPro had a significant impact on the viability of hAECs and hDPSCs at days 2 and 4; however, eDMP revealed a reduction at day 8 (Figure 2A).

### 2.3. Fluorescence Microscopy

Morphologically, the primary culture of the hAECs appeared homogenous with cobblestone-like morphology (Figure 2B–D), whereas hDPSCs were spindle-shaped and considerably smaller (Figure 2E–G). Overall, no relevant medium-dependent changes in cellular morphology were displayed by either cell type.

### 2.4. Cell Adhesion to Dentin

**Figure 3:**
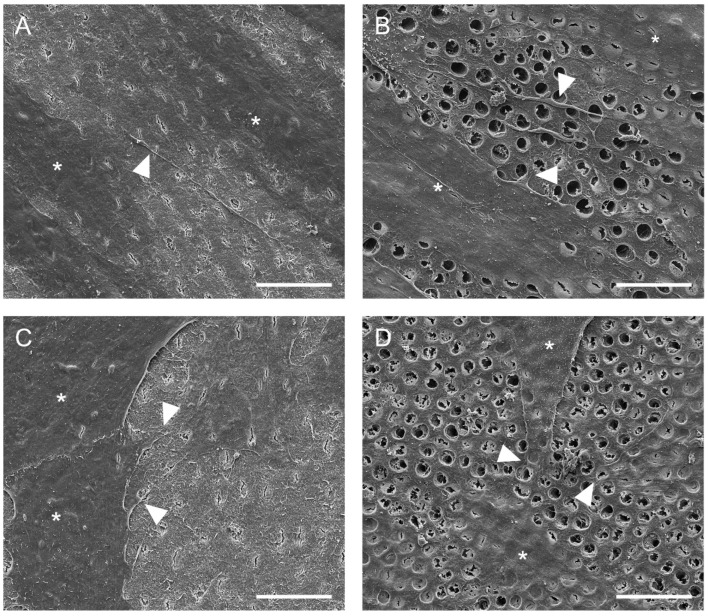
Adhesion of hDPSC and hAECs onto dentin surface. Representative SEM images of dentin surface with hDPSC (**A**,**B**) and hAECs (**C**,**D**) after 48 h (cells marked by asterisks). Cell adhesion and spreading on the surface of dentin was evident with (**B**,**D**) and without (**A**,**C**) EDTA conditioning. Some cytoplasmic processes (arrowheads) were evident in both cell types. (Scale bars: 20 µm).

Representative scanning electron microscopic images of hDPSCs and hAECs on dentin disks are shown in Figure 3. Scanning electron microscope images revealed that hDPSCs (Figure 3A,B) and hAECs (Figure 3C,D) were homogeneously distributed on the dentin. Moreover, both cell types showed adhesion to dentin and spread their processes over the surface in both EDTA-conditioned (Figure 3B,D) and unconditioned (Figure 3A,C) dentin. EDTA-conditioned dentin exhibited a clean dentin surface where dentin tubules were visible, while tubules were covered with a smear layer in unconditioned disks. Both hDPSCs and hAECs extended processes to form cellular contacts (Figure 3B,D). Whereas the hDPSCs adhered to dentin appeared spindle-shaped (Figure 3A,B), the hAECs retained their typical cubic morphology (Figure 3C,D).

### 2.5. Gene Expression

**Figure 4:**
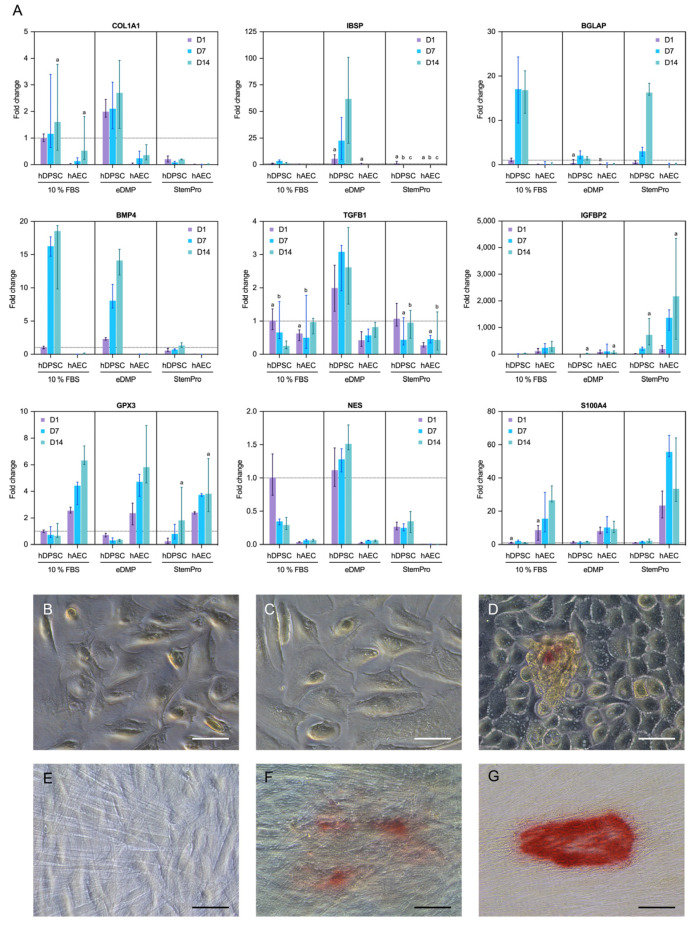
Expression of odontogenic and mineralization-associated genes. Effect of eDMPs and StemPro on expression of odontogenic and mineralization-associated marker genes (*COL1A1*, *BMP4*, *IBSP*, *IGFBP-2*, *NES*, *TGFB1* and *BGLAP*) in hAECs and hDPSCs at days 1, 7 and 14 (**A**). Genes indicative of epithelial–mesenchymal transition (*S100A4*) and protection against oxidative damage (*GPX3*) are also depicted (**A**). Target gene expressions are depicted relative to the untreated control (hDPSCs with 10% FBS at day 1) and median values were calculated from two independent experiments in duplicated samples (*n* = 4). Non-significant differences between hAECs and hDPSCs for each medium and follow-up point are marked with lowercase letters (a, b, c). The effect of eDMPs and StemPro on mineralization of hAECs (**B**–**D**) and hDPSCs (**E**–**G**) using Alizarin Red staining assay. Calcium deposits were evident in hAECs cultured with StemPro (**D**) and hDPCS cultured with eDMPs (**F**) and StemPro (**G**). (Scale bars: 50 µm).

Genes associated with odontoblast differentiation and mineralization (collagen type I alpha 1 chain (*COL1A1)*, bone morphogenetic protein 4 (*BMP4),* integrin binding sialoprotein *(IBSP),* nestin *(NES)* and bone gamma-carboxyglutamate protein or osteocalcin (*BGLAP)*) were either not expressed in hAECs or the expression was significantly downregulated in comparison to the hDPSCs (Figure 4A). However, genes associated with epithelial–mesenchymal transition were upregulated in hAECs compared to hDPSCs. Specifically, the insulin like growth factor binding protein 2 (*IGFBP2*) gene was significantly upregulated in hAECs cultured with StemPro or eDMPs at days 1 and 7, and S100 calcium binding protein A4 (*S100A4*) was considerably upregulated in hAECs at all time points (Figure 4A). Glutathione peroxidase 3 (*GPX3*), a gene associated with the reduction of hydrogen peroxide, which arises from oxidative stress, was significantly upregulated in hAECs in almost all groups and at all time points.

### 2.6. Mineralization

Representative images of the mineralization capability of hAECs and hDPSCs after cultivation with eDMP and StemPro are shown in Figure 4B–G. Calcium deposits were observed in hAECs and hDPSCs cultured with StemPro at day 21 (Figure 4D,G) and in hDPSCs cultured with eDMPs (Figure 4F). Mineralization appeared in the form of small and dense nodules, which were significantly smaller in hAECs. However, no calcium deposits were observed in either cell type cultured in their respective standard media (Figure 4B,E) or in hAECs cultured with eDMPs (Figure 4C).

## 3. Discussion

Revitalization is a promising endodontic therapy for immature necrotic teeth with excellent clinical results [40]. However, the newly generated tissues are reported to be reparative tissues, which show microanatomical deficits, such as the lack odontoblast cells at the dentin–pulp interface [41]. In order to restore the pulp to its original form and function, numerous tissue-engineering-approaches are currently being investigated based on the concepts of cell transplantation as well as cell homing. Due to their pluripotency, hAECs differentiate into various cell types in vivo depending on their local environment. This has been demonstrated by injection of hAECs into the liver and into bone defects and heart tissue and observing the adequate differentiation into functional hepatocytes [42], osteoblasts [43] and cardiomyocytes [44], respectively. To the best of our knowledge, no study has evaluated the odontogenic differentiation of hAECs so far. The results revealed that human hAECs can adhere and spread on dentin and that they are able to mineralize; however, the transition into an odontoblast lineage was not observed.

### 3.1. Cell Adhesion to Dentin

In the context of pulp regeneration, cell attachment to the dentin walls of the root canal is essential [45]. Anatomical and functional restoration of the pulp–dentin complex is only possible with a stable adhesion of the transplanted or recruited stem cells to the collagenous extracellular matrix of dentin. The establishment of an odontoblast layer [27] is necessary as an immunological cellular barrier of the pulp [20] and enables a continuous mineralization in contact with the dentin walls upon the receipt of external stimuli. In the present study, the dentin disks were conditioned with EDTA prior to cell seeding. As a chelator of calcium, it removes the smear layer and provides a clean surface with exposed dentin tubules [46]. Furthermore, its demineralizing effect releases bioactive proteins and growth factors from the dentin extracellular matrix [47] which facilitate stem cell migration, mineralization and odontogenic differentiation [26]. In the clinical situation, EDTA also reverses the deleterious effects of the disinfectant sodium hypochlorite on the survival of stem cells [48], which makes it a crucial irrigation step in the current recommendations for revitalization procedures [49]. To investigate the interaction of hAECs with dentin, cells were isolated from human placentas and cultured directly on dentin disks for 48 h. Scanning electron microscope images revealed that hAECs were able to adhere and spread on dentin in a similar fashion to the dental pulp stem cells irrespective of EDTA-conditioning. As expected, the remaining smear layer did not affect the cells’ survival; however, unconditioned dentin did not provide the necessary access to the tubules.

### 3.2. Mineralization

After proper adhesion of the cells to the dentin matrix, they are expected to differentiate into an odontogenic or osteogenic phenotype [27] and to start to secrete a mineralized matrix. While osteogenic differentiation was induced with StemPro as commercial differentiation medium, development into an odontogenic lineage was promoted by the addition of extracted human eDMPs, as has been carried out and described in previous studies [26,50]. Alizarin Red staining after 21 days revealed that human amnion epithelial cells cultured with StemPro medium were able to produce calcium deposits, confirming the basic mineralization capacity of these cells, as shown previously [43,51]; however, the calcification nodules were small and scarce compared to those of hDPSCs. Morphologically, a slight enlargement of the hAECs was observed after induced osteogenesis, which did not mirror previous investigations that reported a two-to-three-fold expansion of cell bodies following osteogenic differentiation [30]. Overall, hAECs appeared not to respond to osteogenic culture medium as well as was described in other studies [51,52]. Importantly, hAECs cultured with 500 pg/mL of eDMPs did not reveal any calcium deposits [26], in contrast to hDPSCs, which allows the conclusion that hAECs are not responsive to eDMPs in the tested concentrations either.

### 3.3. Cell Differentiation

StemPro osteogenic differentiation medium and eDMPs induce osteogenic and odontogenic differentiation of mesenchymal stem cells, respectively [26]. In previous studies, hAECs cultured with standard osteogenic medium [43,51], i.e., medium containing β-glycerophosphate and dexamethasone, showed an increase in alkaline phosphatase activity as well as the expression of bone-related genes [43,51]. As far as we know, the effect of eDMPs and StemPro on amniotic epithelial stem cells has not been investigated so far. In accordance with existing research, hDPSCs were able to differentiate into an odontogenic cell type [53,54]. This was shown by the upregulation of typical marker genes for both osteogenic and odontogenic lineage: *COL1A1, IBSP, BGLAP, BMP4* and transforming growth factor beta 1 (*TGF-β1*) in qRT-PCR. In this context, COL1A1 is classified as a marker for early mineralization [55,56,57]. IBSP is secreted during crystallization [58,59]. *BGLAP* can actively bind calcium and is therefore a late marker for mineralization [26,56,58,60,61]. *BMP4* is known to stimulate odontogenesis and bone formation [62,63,64]. *TGF-β1* is expressed by odontoblasts during maturation and dentinogenesis [65]. Furthermore, the upregulation of the dental specific marker *NES* confirms a differentiation of the hDPSCs along the odontogenic cell line [59,66]. To evaluate the odontoblastic transition of hAECs cultured with eDMPs, the expression of genes related to odontoblast differentiation and mineralization, including *COL1A1, BMP4, IBSP,* *IGFBP2, NES* and *BGLAP* (osteocalcin) [67], was analyzed and compared with hDPSCs. We expected to see similar results for hAECs in eDMP; however, the hAECs did not show any significant upregulation of mineralization markers under odontogenic culture conditions. We therefore conclude that, under the settings of this study, the hAECs were unable to differentiate into an odontogenic phenotype.

### 3.4. Epithelial–Mesenchymal Transition

A possible explanation for the compromised differentiation displayed by the otherwise pluripotent hAECs could be that they underwent epithelial–mesenchymal transition (EMT), which has been associated with a reduced osteogenic differentiability [68]. During this process the epithelial cells lose their characteristics, such as polarization or cell–cell connections, and change their phenotype to that of mesenchymal cells. EMT can be physiological, e.g., during embryonic development, inflammation, wound healing or fibrosis, but is also part of pathological processes, such as tumor progression or oncogenesis. Interestingly, it has even been observed to occur in cell culture [69,70]. Reportedly, freshly isolated hAECs do not express mesenchymal surface markers, such as CD105 and CD44, but display primarily epithelial markers, such as CD49f and CD326 [29,71,72,73]. However, cells in this study also expressed CD105 and CD44, which is in line with research concerning cultured and expanded hAECs [74,75,76,77]. This increasing change in phenotype over the cultivation period has previously been described [68] and can also be seen as an indication that the cells undergo EMT. Furthermore, S100A4, a calcium-binding protein also called fibroblast specific protein 1, has been described as a marker for this transition [78]. *IGFBP2*, which was upregulated by hAECs up to 4000-fold in comparison to the expression in DPSC, can also be classified as an EMT marker [79]. A way to induce the EMT process in vitro can be the addition of epidermal growth factor (EGF) and TGF-β, both of which were necessarily in the media [80]. Autocrine TGF-β production can also stimulate this process [81]. An additional explanation for why EMT is occurring could be unintentional selection of the mesenchymal phenotype by the culture protocol. Cells without CD44 expression are more likely to detach from the culture flask and thereby be removed during medium change [68]. However, the change from an epithelial to a mesenchymal phenotype is described as being accompanied by a change in morphology [68], which was not observed in our experiment. Further research needs to be undertaken comparing the differentiability of freshly isolated and expanded hAECs.

### 3.5. Impact of Culture Conditions

Glutathione peroxidase 3 (*GPX3*) aims at the reduction of hydrogen peroxide, which may arise from oxidative stress in cell metabolism. In this case, increased *GPX3* levels might accompany stressful culture conditions or trypsination of the cells, which is commonly paraphrased as “culture shock” [82,83]. Reportedly, hAECs are a highly sensitive cell type and quite challenging in in vitro culture [84]. Their viability was significantly lower compared to hDPSCs over all time points and EGF was essential to provide a more physiological environment; however, analogously to the observations by other research groups, the cells did not thrive outside their specific stem cell niche [38]. The upregulation of *GPX3* by hAECs, in combination with the reduced metabolic activity in eDMP, as indicated by the MTT assay, could indicate unfavorable culture conditions that might affect differentiation. This assumption is to be verified in further experiments by, e.g., determining intracellular reactive oxygen species (ROS) or antioxidative enzymes.

## 4. Materials and Methods

### 4.1. Isolation and Characterization of hAECs

Human placentas were obtained from caesarean deliveries of healthy donors with informed consent and the approval of the Bioethical Commission of the University of Barcelona, Spain (No.: IRB00003099). The placentas were transported to the laboratory for further processing in sterile saline at 4 °C. The hAECs were isolated in a laminar flow cabinet following a previously published protocol [30]. Briefly, the amnion was detached from the underlying chorion and washed with 200 mL of Ringer’s acetate solution (pH 6.5; Baxter, Deerfield, MA, USA) for up to 10 min and 200 mL of PBS (PBS, Biochrom, Berlin, Germany) to remove all blood particles. Subsequently, 2–3 g portions of amnion were digested in Falcon tubes with 20 mL of 10× TryPLE solution (Life Technologies, Gaithersburg, USA) on a shaker (35 rpm) at 37 °C for 30 min. The membrane underwent a second digestion step with fresh digestion solution. Cells from both digestions were centrifuged at 300× *g* for 10 min and suspended in DMEM (DMEM, high glucose; Life Technologies, Gaithersburg, MD, USA) with 10% FBS, 100 U/mL penicillin and 100 µg/mL streptomycin, 2 mM L-glutamine, 1 mM sodium pyruvate and 10 ng/mL of epidermal growth factor (EGF; StemCell Technologies, Vancouver, BC, Canada). This medium containing EGF, which is an essential supplement for hAEC growth, is referred to as DMEM 10% FBS in the following text. Cells from passage 2 were used in all experiments and cultured at 37 °C and 5% CO_2_. To assess the effectiveness of the isolation procedure, amniotic tissue before digestion as well as after the first and second digestion step was fixed in formalin for 2 h and processed for histology. Histological processing and HE staining was performed according to a previously published protocol [85]. Furthermore, flow cytometry was conducted to evaluate the antigen profile of the isolated cells. Immediately after isolation, hAECs were seeded in T75 flasks and cultured to 80% confluence with DMEM 10% FBS. Finally, a suspension of 2 × 10^5^ cells in 81 µL was incubated with 2 µL of mouse anti-human CD44 (APC; 560890, BD Biosciences, San Jose, CA, USA), 5 µL of mouse anti-human CD105 (PerCP-CY 5.5; 562245, BD Biosciences, San Jose, CA, USA), 8 µL of anti-CD326 (FITC; 347197, BD Biosciences, San Jose, CA, USA) and 4 µL of rat anti-human CD49f (PE; 561894, BD Biosciences, San Jose, CA, USA). Flow cytometry was conducted with at least 10,000 events per sample (FACSCant, BD Biosciences, San Jose, CA, USA) and data was analyzed with FlowJo (version 10.8, BD Biosciences, San Jose, CA, USA). Cells from two different donors were investigated in triplicate and median values with 25–75% percentiles were computed (*n* = 6).

### 4.2. Isolation and Characterization of hDPSCs

Human dental pulp stem cells were isolated from human third molars and cultured as described previously [86]. Dental pulp stem cells were maintained in αMEM supplemented with 100 U/mL penicillin, 100 µg/mL streptomycin and 10% FBS. Finally, to ensure mesenchymal stem cell character, the cells were sorted for the surface antigen STRO-1using the MACS-System (magnetic-activated cell sorting; Miltenyi Biotec, Bergisch Gladbach, Germany). Furthermore, mesenchymal stem cell antigens, following Dominici et al. [87], were determined in accordance with a previous work [50].

### 4.3. Extraction of Dentin Matrix Proteins (eDMPs)

Human caries-free third molars were collected from donors (15–25 years old) after informed consent and with approval by an appropriate review board at the University of Regensburg (No.: 19-1327-101; Faculty of Medicine, University of Regensburg, Regensburg, Germany). eDMPs were extracted from human teeth according to a validated protocol [26]. Briefly, dentin was pulverized (Mixer Mill MM 200, RETSCH, Haan, Germany) after removal of the enamel, cementum and pulp. Dentin powder was suspended in 10% EDTA (AppliChem, Darmstadt, Germany) and the solution was purified with syringe filters (1.2, 0.45, and 0.2 µm Acrodisc Syringe Filters with Supor Membrane; Pall Corporation, Port Washington, WI, USA) and enriched by centrifugal filtration with a molecular weight cut-off of at 3000 Da (Amicon Ultra-15 3K; Merck Millipore, Billerica, MA, USA). The solvent was exchanged for phosphate-buffered saline (PBS without Ca^2+^, Mg^2+^; Biochrom, Berlin, 137 Germany). Finally, TGF-β1 was quantified as a representative growth factor (Quantikine 138 ELISA Kit; R&D Systems Inc., Minneapolis, MN, USA) to facilitate standardized supplementation to culture media.

### 4.4. Cell Viability

To quantify the cell viability, hAECs (3.2 × 10^3^ cells/well) and hDPSCs (3.2 × 10^3^ cells/well) were seeded in 96-well plates to reach 80% confluency. The hAECs were exposed to the following media: (i) DMEM 10% FBS; (ii) DMEM 10% FBS and 500 pg/mL eDMPs; and (iii) osteogenic differentiation medium with 10 ng/mL EGF (StemPro Osteogenesis Differentiation Kit; Thermo Fisher Scientific, Waltham, MA, USA). Likewise, hDPSCs were cultured in (i) α-MEM with 10% FBS, (ii) α-MEM with 10% FBS and 500 pg/mL eDMPs and (iii) osteogenic differentiation medium (StemPro Osteogenesis Differentiation Kit; Thermo Fisher Scientific, Waltham, MA, USA). MTT assays were performed after 2, 4 and 8 days. The cells were incubated with 100 µL/well of a 0.5 mg/mL MTT solution (Thiazolyl Blue Tetrazolium Bromide; Sigma-Aldrich, Saint Louis, MO, USA) for 60 min at 37 °C and 5% CO_2_. Subsequently, the dye was dissolved in 200 µL/well of dimethyl sulfoxide (DMSO; Merck Millipore, Billerica, MA, USA) on a shaker (540 rpm) at room temperature for 10 min. Optical density readings were performed on a microplate reader at λ = 540 nm (Infinite 200; Tecan, Männedorf, Switzerland) and the results were summarized as median values with 25–75% percentiles (*n* = 8).

### 4.5. Fluorescence Microscopy

To evaluate morphological changes induced by the three different types of media, hAECs (7.5 × 10^3^ cells/well) and hDPSCs (5 × 10^3^ cells/well) were seeded on coverslips in 24-well plates and cultured as described above. After 7 days, cells were fixed with 4% formalin (10 min), permeabilized with 0.1% Triton X (5 min) and stained with phalloidin (30 min) and DAPI (1 min). Coverslips were mounted on slides (ProLong Glass Antifade Mountant, Thermo Fisher Scientific, Waltham, USA) and imaged on a ZEISS microscope (Axio Vert.A1, Carl Zeiss Microscopy, Jena, Germany) with the ZEISS Axiocam 503 color camera (Carl Zeiss Microscopy, Jena, Germany). Images with filters for blue (Carl Zeiss Microscopy, Jena, Germany) and red fluorescence (Set 43 and Set 49, Carl Zeiss Microscopy, Jena, Germany) in place were taken independently and digitally superimposed. ZEN software was used for microscopy and imaging (version 3.1, Carl Zeiss Microscopy, Jena, Germany).

### 4.6. Cell Adhesion to Dentin

The adherence and phenotypic changes of hAECs and hDPSCs seeded on dentin disks were evaluated by scanning electron microscopy. Dentin disks of 0.2 mm thickness were obtained from the crown of human molars. They were optionally rinsed in 10% EDTA for 15 min and washed with distilled water afterwards. Subsequently, hAECs and hDPSCs (3.8 × 10^4^ cells/well) were seeded on the dentin disks in 24-well plates. The hAECs were cultured in DMEM with 10% FBS and DPSCs in α-MEM with 10% FBS for 48 h (37 °C, 5% CO_2_). Samples were fixed with 2.5% glutaraldehyde in 0.1 M Sørensen’s phosphate buffer for 30 min and analyzed on a FEI Quanta 400 environmental scanning electron microscope (SEM) with a field emitter and an Everhart–Thornley detector at 4.0 kV and high-vacuum conditions (FEI Europe B.V., Eindhoven, The Netherlands).

### 4.7. Gene Expression

Cultures were established with hAECs (7.5 × 10^4^ cells/well) and hDPSCs (4.9 × 10^4^ cells/well) in 12-well plates, as described above. After 1, 7 and 14 days, mRNA was extracted using the RNeasy Mini Kit (Qiagen, Hilden, Germany) and quantified spectrophotometrically (NanoDrop 2000, Thermo Fisher Scientific, Waltham, USA). Then, 500 ng of nucleic acids were transcribed into cDNA (Omniscript RT Kit, Qiagen, Hilden, Germany) using oligo-dT primers (Qiagen, Hilden, Germany). To assess the effect of eDMP and StemPro on gene expression, qRT-PCR was performed using the TaqMan Fast Advanced Master 188 Mix (4444557, Applied Biosystems, Thermo Fisher Scientific, Waltham, MA, USA) and probes for the following genes: collagen type I alpha 1 (COL1A1; Hs00164004_m1), integrin binding sialoprotein (IBSP; Hs00913377_m1), bone gamma-carboxyglutamate protein (BGLAP; Hs01587814_g1), bone morphogenic protein 4 (BMP4; Hs00370078_m1), transforming growth factor beta 1 (TGFB1; Hs00998133_m1), nestin (NES; Hs04187831_g1), insulin-like growth factor binding protein 2 (IGFBP2; Hs01040719_m1), S100 calcium binding protein A4 (S100A4; Hs00243202_m1), glutathione peroxidase 3 (GPX3; Hs01078668_m1) and 40S ribosomal protein S18 (RPS18; Hs99999901_s1) as the housekeeping gene. Finally, measurements for all target genes were normalized to RPS18 and related to hDPSCs cultured in 10% by the comparative CT method (ΔΔCT) [88]. Medians with 25–75% percentiles were calculated on the basis of two experiments with cells from different donors (*n* = 4).

### 4.8. Mineralization

To visualize calcium deposition, hAECs (3.7 × 10^4^ cells/well) and hDPSCs (2.4 × 10^4^ cells/well) were seeded in 24-well plates to reach 80% confluency. Both cell types were cultured according to the previously described groups. After 21 days, cells were fixed with formalin for 10 min and incubated with 40 mM alizarin (Alizarin Red S, Carl Roth, Karlsruhe, Germany) at pH 4.2 and room temperature for 30 min. Images were taken with an inverted microscope (Axio Vert.A1, Carl Zeiss Microscopy GmbH, Jena, Germany).

### 4.9. Statistical Analysis

Data were treated nonparametrically and pairwise Mann–Whitney U tests were conducted at a significance level of α = 0.05. Statistical analysis was performed, comparing hAECs and hDPSCs for each medium for each follow-up point. All statistical analyses were computed with GraphPad Prism 9 (GraphPad Software, La Jolla, CA, USA) and non-statistical significance (*p* > 0.05) was indicated in the respective figures by lowercase letters.

## 5. Conclusions

Human amnion epithelial cells can adhere and spread on dentin and are able to differentiate and mineralize. Nevertheless, hAECs failed to reveal an odontoblastic transition under in vitro conditions. Even if hAECs show great promise in other regenerative applications, they do not seem to be a feasible alternative stem cell source for dental pulp tissue engineering. In addition to the difficult culture behavior, their cellular reactions are difficult to control by signaling molecules and are not as reliable as, for example, mesenchymal stem cells from pulp, which was particularly evident in EMT. Thus, the advantages of hAECs in terms of pluripotency do not come into play and their advantages in terms of high availability can presumably not be used.

## Data Availability

Data is contained within the article.

## References

[1] Murray P., Garcia-Godoy F., Hargreaves K.M. (2007). Regenerative Endodontics: A Review of Current Status and a Call for Action. J. Endod..

[2] Cvek M. (1992). Prognosis of luxated non-vital maxillary incisors treated with calcium hydroxide and filled with gutta-percha. A retrospective clinical study. Dent. Traumatol..

[3] Tong H.J., Rajan S., Bhujel N., Kang J., Duggal M., Nazzal H. (2017). Regenerative Endodontic Therapy in the Management of Nonvital Immature Permanent Teeth: A Systematic Review—Outcome Evaluation and Meta-analysis. J. Endod..

[4] Lin J., Zeng Q., Wei X., Zhao W., Cui M., Gu J., Lu J., Yang M., Ling J. (2017). Regenerative Endodontics Versus Apexification in Immature Permanent Teeth with Apical Periodontitis: A Prospective Randomized Controlled Study. J. Endod..

[5] Jiang X., Liu H., Peng C. (2017). Clinical and Radiographic Assessment of the Efficacy of a Collagen Membrane in Regenerative Endodontics: A Randomized, Controlled Clinical Trial. J. Endod..

[6] Nazzal H., Kenny K., Altimimi A., Kang J., Duggal M.S. (2018). A prospective clinical study of regenerative endodontic treatment of traumatized immature teeth with necrotic pulps using bi-antibiotic paste. Int. Endod. J..

[7] Zhu W., Zhu X., Huang G.T.-J., Cheung G.S.P., Dissanayaka W., Zhang C. (2013). Regeneration of dental pulp tissue in immature teeth with apical periodontitis using platelet-rich plasma and dental pulp cells. Int. Endod. J..

[8] Meschi N., Hilkens P., Lambrichts I., Van den Eynde K., Mavridou A., Strijbos O., De Ketelaere M., Van Gorp G., Lambrechts P. (2015). Regenerative endodontic procedure of an infected immature permanent human tooth: An immunohistological study. Clin. Oral Investig..

[9] Wang X., Thibodeau B., Trope M., Lin L.M., Huang G.T.-J. (2010). Histologic Characterization of Regenerated Tissues in Canal Space after the Revitalization/Revascularization Procedure of Immature Dog Teeth with Apical Periodontitis. J. Endod..

[10] Bucchi C., Marcé-Nogué J., Galler K.M., Widbiller M. (2019). Biomechanical performance of an immature maxillary central incisor after revitalization: A finite element analysis. Int. Endod. J..

[11] Orti V., Collart-Dutilleul P.-Y., Piglionico S., Pall O., Cuisinier F., Panayotov I. (2018). Pulp Regeneration Concepts for Nonvital Teeth: From Tissue Engineering to Clinical Approaches. Tissue Eng. Part B Rev..

[12] Galler K.M., Widbiller M. (2017). Perspectives for Cell-homing Approaches to Engineer Dental Pulp. J. Endod..

[13] He L., Zhong J., Gong Q., Cheng B., Kim S.G., Ling J., Mao J.J. (2017). Regenerative Endodontics by Cell Homing. Dent. Clin..

[14] Nakashima M., Iohara K., Murakami M., Nakamura H., Sato Y., Ariji Y., Matsushita K. (2017). Pulp regeneration by transplantation of dental pulp stem cells in pulpitis: A pilot clinical study. Stem Cell Res. Ther..

[15] Ducret M., Fabre H., Celle A., Mallein-Gerin F., Perrier-Groult E., Alliot-Licht B., Farges J.-C. (2017). Current challenges in human tooth revitalization. Bio.-Med. Mater. Eng..

[16] Huang G.T.-J. (2011). Dental pulp and dentin tissue engineering and regeneration advancement and challenge. Front. Biosci..

[17] Couve E., Osorio R., Schmachtenberg O. (2014). Reactionary Dentinogenesis and Neuroimmune Response in Dental Caries. J. Dent. Res..

[18] Couve E., Osorio R., Schmachtenberg O. (2013). The Amazing Odontoblast. J. Dent. Res..

[19] Farges J.-C., Keller J.-F., Carrouel F., Durand S.H., Romeas A., Bleicher F., Lebecque S., Staquet M.-J. (2009). Odontoblasts in the dental pulp immune response. J. Exp. Zoöl. Part B Mol. Dev. Evol..

[20] Farges J.-C., Alliot-Licht B., Renard E., Ducret M., Gaudin A., Smith A.J., Cooper P.R. (2015). Dental Pulp Defence and Repair Mechanisms in Dental Caries. Mediat. Inflamm..

[21] Farges J.-C., Bellanger A., Ducret M., Aubert-Foucher E., Richard B., Alliot-Licht B., Bleicher F., Carrouel F. (2015). Human odontoblast-like cells produce nitric oxide with antibacterial activity upon *TLR2* activation. Front. Physiol..

[22] Farges J.-C., Carrouel F., Keller J.-F., Baudouin C., Msika P., Bleicher F., Staquet M.-J. (2011). Cytokine production by human odontoblast-like cells upon Toll-like receptor-2 engagement. Immunobiology.

[23] Itoh Y., Sasaki J.I., Hashimoto M., Katata C., Hayashi M., Imazato S. (2018). Pulp Regeneration by 3-dimensional Dental Pulp Stem Cell Constructs. J. Dent. Res..

[24] Iohara K., Imabayashi K., Ishizaka R., Watanabe A., Nabekura J., Ito M., Matsushita K., Nakamura H., Nakashima M. (2011). Complete Pulp Regeneration after Pulpectomy by Transplantation of CD105+ Stem Cells with Stromal Cell-Derived Factor-1. Tissue Eng. Part A.

[25] Xuan K., Li B., Guo H., Sun W., Kou X., He X., Zhang Y., Sun J., Liu A., Liao L. (2018). Deciduous autologous tooth stem cells regenerate dental pulp after implantation into injured teeth. Sci. Transl. Med..

[26] Widbiller M., Eidt A., Lindner S.R., Hiller K.-A., Schweikl H., Buchalla W., Galler K.M. (2018). Dentine matrix proteins: Isolation and effects on human pulp cells. Int. Endod. J..

[27] Galler K.M., D’Souza R., Federlin M., Cavender A.C., Hartgerink J., Hecker S., Schmalz G. (2011). Dentin Conditioning Codetermines Cell Fate in Regenerative Endodontics. J. Endod..

[28] Miki T. (2018). Stem cell characteristics and the therapeutic potential of amniotic epithelial cells. Am. J. Reprod. Immunol..

[29] Gramignoli R., Srinivasan R.C., Kannisto K., Strom S.C. (2016). Isolation of Human Amnion Epithelial Cells According to Current Good Manufacturing Procedures. Curr. Protoc. Stem Cell Biol..

[30] Ilancheran S., Michalska A., Peh G., Wallace E.M., Pera M., Manuelpillai U. (2007). Stem Cells Derived from Human Fetal Membranes Display Multilineage Differentiation Potential. Biol. Reprod..

[31] Gronthos S., Mankani M., Brahim J., Robey P.G., Shi S. (2000). Postnatal human dental pulp stem cells (DPSCs) in vitro and in vivo. Proc. Natl. Acad. Sci. USA.

[32] Yang P.-J., Yuan W.-X., Liu J., Li J.-Y., Tan B., Qiu C., Zhu X.-L., Qiu C., Lai D.-M., Guo L.-H. (2018). Biological characterization of human amniotic epithelial cells in a serum-free system and their safety evaluation. Acta Pharmacol. Sin..

[33] Nemr W., Bashandy M., Araby E., Khamiss O. (2017). Molecular displaying of differential immunoresponse to various infections of amniotic epithelia. Am. J. Reprod. Immunol..

[34] Motedayyen H., Fathi F., Fasihi-Ramandi M., Taheri R.A. (2018). The effect of lipopolysaccharide on anti-inflammatory and pro-inflammatory cytokines production of human amniotic epithelial cells. Reprod. Biol..

[35] Zhu D., Muljadi R., Chan S.T., Vosdoganes P., Lo C., Mockler J.C., Wallace E., Lim R. (2015). Evaluating the Impact of Human Amnion Epithelial Cells on Angiogenesis. Stem Cells Int..

[36] Miki T., Lehmann T., Cai H., Stolz D.B., Strom S.C. (2005). Stem Cell Characteristics of Amniotic Epithelial Cells. Stem Cells.

[37] Lim R., Hodge A., Moore G., Wallace E.M., Sievert W. (2017). A Pilot Study Evaluating the Safety of Intravenously Administered Human Amnion Epithelial Cells for the Treatment of Hepatic Fibrosis. Front. Pharmacol..

[38] Miki T., Strom S.C. (2006). Amnion-derived pluripotent/multipotent stem cells. Stem Cell Rev. Rep..

[39] Bajaj M., Soni A.J. (2019). Revascularization of a Nonvital, Immature Permanent Tooth Using Amniotic Membrane: A Novel Approach. Int. J. Clin. Pediatr. Dent..

[40] Chrepa V., Joon R., Austah O., Diogenes A., Hargreaves K.M., Ezeldeen M., Ruparel N.B. (2020). Clinical Outcomes of Immature Teeth Treated with Regenerative Endodontic Procedures—A San Antonio Study. J. Endod..

[41] Becerra P., Ricucci D., Loghin S., Gibbs J.L., Lin L.M. (2014). Histologic Study of a Human Immature Permanent Premolar with Chronic Apical Abscess after Revascularization/Revitalization. J. Endod..

[42] Lin J.S., Zhou L., Sagayaraj A., Jumat N.H.B., Choolani M., Chan J.K.Y., Biswas A., Wong P.C., Lim S.G., Dan Y.Y. (2015). Hepatic differentiation of human amniotic epithelial cells and in vivo therapeutic effect on animal model of cirrhosis. J. Gastroenterol. Hepatol..

[43] Mattioli M., Gloria A., Turriani M., Mauro A., Curini V., Russo V., Tetè S., Marchisio M., Pierdomenico L., Berardinelli P. (2011). Stemness characteristics and osteogenic potential of sheep amniotic epithelial cells. Cell Biol. Int..

[44] Fang C.-H., Jin J., Joe J.-H., Song Y.-S., So B.-I., Lim S.M., Cheon G.J., Woo S.-K., Ra J.-C., Lee Y.-Y. (2012). In Vivo Differentiation of Human Amniotic Epithelial Cells into Cardiomyocyte-Like Cells and Cell Transplantation Effect on Myocardial Infarction in Rats: Comparison with Cord Blood and Adipose Tissue-Derived Mesenchymal Stem Cells. Cell Transplant..

[45] Galler K.M., Widbiller M., Buchalla W., Eidt A., Hiller K.-A., Hoffer P.C., Schmalz G.H. (2016). EDTA conditioning of dentine promotes adhesion, migration and differentiation of dental pulp stem cells. Int. Endod. J..

[46] Morago A., Ruiz-Linares M., Ferrer-Luque C.M., Baca P., Archilla A.R., Arias-Moliz M.T. (2019). Dentine tubule disinfection by different irrigation protocols. Microsc. Res. Tech..

[47] Galler K.M., Buchalla W., Hiller K.-A., Federlin M., Eidt A., Schiefersteiner M., Schmalz G. (2015). Influence of Root Canal Disinfectants on Growth Factor Release from Dentin. J. Endod..

[48] Martin D.E., de Almeida J.F.A., Henry M.A., Khaing Z., Schmidt C.E., Teixeira F.B., Diogenes A. (2014). Concentration-dependent Effect of Sodium Hypochlorite on Stem Cells of Apical Papilla Survival and Differentiation. J. Endod..

[49] Galler K.M., Krastl G., Simon S., Van Gorp G., Meschi N., Vahedi B., Lambrechts P. (2016). European Society of Endodontology position statement: Revitalization procedures. Int. Endod. J..

[50] Widbiller M., Eidt A., Wölflick M., Lindner S.R., Schweikl H., Hiller K.-A., Buchalla W., Galler K.M. (2018). Interactive effects of LPS and dentine matrix proteins on human dental pulp stem cells. Int. Endod. J..

[51] Si J., Zhang J., Dai J., Yu D., Yu H., Shi J., Wang X., Shen S.G.F., Guo L. (2014). Osteogenic Differentiation of Human Amniotic Epithelial Cells and Its Application in Alveolar Defect Restoration. Stem Cells Transl. Med..

[52] Fatimah S.S., Ng S.L., Chua K.H., Hayati A.R., Tan A.E., Tan G.C. (2010). Value of human amniotic epithelial cells in tissue engineering for cornea. Hum. Cell.

[53] Huang G.T.-J., Shagramanova K., Chan S.W. (2006). Formation of Odontoblast-Like Cells from Cultured Human Dental Pulp Cells on Dentin In Vitro. J. Endod..

[54] Liu G., Xu G., Gao Z., Liu Z., Xu J., Wang J., Zhang C., Wang S. (2016). Demineralized Dentin Matrix Induces Odontoblastic Differentiation of Dental Pulp Stem Cells. Cells Tissues Organs.

[55] Braut A., Kollar E.J., Mina M. (2003). Analysis of the odontogenic and osteogenic potentials of dental pulp in vivo using a *Col1a1-2.3-GFP* transgene. Int. J. Dev. Biol..

[56] Huang W., Yang S., Shao J., Li Y.P. (2007). Signaling and transcriptional regulation in osteoblast commitment and differentiation. Front. Biosci..

[57] Kaneto C.M., Lima P.S.P., Zanette D.L., Oliveira T.Y.K., Pereira F.D.A., Lorenzi J.C.C., Dos Santos J.L., Prata K.L., Neto J.M.P., De Paula F.J.A. (2016). Osteoblastic differentiation of bone marrow mesenchymal stromal cells in Bruck Syndrome. BMC Med Genet..

[58] Simon S., Smith A., Lumley P., Berdal A., Smith G., Finney S., Cooper P. (2009). Molecular characterization of young and mature odontoblasts. Bone.

[59] Smith A.J., Scheven B.A., Takahashi Y., Ferracane J.L., Shelton R.M., Cooper P.R. (2012). Dentine as a bioactive extracellular matrix. Arch. Oral Biol..

[60] Chen L., Jacquet R., Lowder E., Landis W.J. (2015). Refinement of collagen–mineral interaction: A possible role for osteocalcin in apatite crystal nucleation, growth and development. Bone.

[61] Dacic S., Kalajzic I., Visnjic D., Lichtler A.C., Rowe D.W. (2001). Col1a1-Driven Transgenic Markers of Osteoblast Lineage Progression. J. Bone Miner. Res..

[62] Nakashima M., Nagasawa H., Yamada Y., Reddi A.H. (1994). Regulatory Role of Transforming Growth Factor-β, Bone Morphogenetic Protein-2, and Protein-4 on Gene Expression of Extracellular Matrix Proteins and Differentiation of Dental Pulp Cells. Dev. Biol..

[63] Asgary S., Nazarian H., Khojasteh A., Shokouhinejad N. (2014). Gene Expression and Cytokine Release during Odontogenic Differentiation of Human Dental Pulp Stem Cells Induced by 2 Endodontic Biomaterials. J. Endod..

[64] MacDougall M.J., Javed A., Bronner F., Farach-Carson M.C., Roach H.T. (2010). Dentin and Bone: Similar Collagenous Mineralized Tissues. Bone and Development.

[65] Niwa T., Yamakoshi Y., Yamazaki H., Karakida T., Chiba R., Hu J.C.C., Nagano T., Yamamoto R., Simmer J.P., Margo-lis H.C. (2018). The dynamics of TGF-β in dental pulp, odontoblasts and dentin. Sci. Rep..

[66] About I., Laurent-Maquin D., Lendahl U., Mitsiadis T.A. (2000). Nestin Expression in Embryonic and Adult Human Teeth under Normal and Pathological Conditions. Am. J. Pathol..

[67] Dds M.W., Bucchi C., Rosendahl A., Spanier G., Buchalla W., Galler K.M. (2019). Isolation of primary odontoblasts: Expectations and limitations. Aust. Endod. J..

[68] Stadler G., Hennerbichler S., Lindenmair A., Peterbauer A., Hofer K., van Griensven M., Gabriel C., Redl H., Wolbank S. (2008). Phenotypic shift of human amniotic epithelial cells in culture is associated with reduced osteogenic differentiation in vitro. Cytotherapy.

[69] Okada H., Danoff T.M., Kalluri R., Neilson E.G. (1997). Early role of *Fsp1* in epithelial-mesenchymal transformation. Am. J. Physiol. Physiol..

[70] Hay E.D. (1995). An Overview of Epithelio-Mesenchymal Transformation. Cells Tissues Organs.

[71] Pratama G., Vaghjiani V., Tee J.Y., Liu Y.H., Chan J., Tan C., Murthi P., Gargett C., Manuelpillai U. (2011). Changes in Culture Expanded Human Amniotic Epithelial Cells: Implications for Potential Therapeutic Applications. PLoS ONE.

[72] Tabatabaei M., Mosaffa N., Nikoo S., Bozorgmehr M., Ghods R., Kazemnejad S., Rezania S., Keshavarzi B., Arefi S., Ramezani-Tehrani F. (2014). Isolation and partial characterization of human amniotic epithelial cells: The effect of tryp-sin. Avicenna. J. Med. Biotechnol..

[73] Murphy S.V., Kidyoor A., Reid T., Atala A., Wallace E.M., Lim R. (2014). Isolation, Cryopreservation and Culture of Human Amnion Epithelial Cells for Clinical Applications. J. Vis. Exp..

[74] De Coppi P., Atala A. (2019). Stem Cells from the Amnion. Principles of Regenerative Medicine.

[75] Portmann-Lanz C.B., Schoeberlein A., Huber A., Sager R., Malek A., Holzgreve W., Surbek D.V. (2006). Placental mesenchymal stem cells as potential autologous graft for pre- and perinatal neuroregeneration. Am. J. Obstet. Gynecol..

[76] Wolbank S., Peterbauer A., Fahrner M., Hennerbichler S., van Griensven M., Stadler G., Redl H., Gabriel C. (2007). Dose-Dependent Immunomodulatory Effect of Human Stem Cells from Amniotic Membrane: A Comparison with Human Mesenchymal Stem Cells from Adipose Tissue. Tissue Eng..

[77] Toda A., Okabe M., Yoshida T., Nikaido T. (2007). The Potential of Amniotic Membrane/Amnion-Derived Cells for Regeneration of Various Tissues. J. Pharmacol. Sci..

[78] Ambartsumian N., Klingelhöfer J., Grigorian M., Heizmann C.W. (2019). The Multifaceted S100A4 Protein in Cancer and Inflammation. Calcium Binding Proteins of the EF-Hand Superfamily.

[79] Li T., Forbes M.E., Fuller G.N., Li J., Yang X., Zhang W. (2020). *IGFBP2*: Integrative hub of developmental and oncogenic signaling network. Oncogene.

[80] Zavadil J., Böttinger E.P. (2005). TGF-β and epithelial-to-mesenchymal transitions. Oncogene.

[81] Alcaraz A., Mrowiec A., Insausti C.L., García-Vizcaíno E.M., Ruiz-Canada C., López-Martínez C., Moraleda J.M., Nicolás F.J. (2013). Autocrine TGF-β Induces Epithelial to Mesenchymal Transition in Human Amniotic Epithelial Cells. Cell Transplant..

[82] Halliwell B. (2003). Oxidative stress in cell culture: An under-appreciated problem?. FEBS Lett..

[83] Stolzing A., Sethe S., Scutt A.M. (2006). Stressed Stem Cells: Temperature Response in Aged Mesenchymal Stem Cells. Stem Cells Dev..

[84] Terada S., Matsuura K., Enosawa S., Miki M., Hoshika A., Suzuki S., Sakuragawa N. (2000). Inducing Proliferation of Human Amniotic Epithelial (HAE) Cells for Cell Therapy. Cell Transplant..

[85] Widbiller M., Rothmaier C., Saliter D., Wölflick M., Rosendahl A., Buchalla W., Schmalz G., Spruss T., Galler K.M. (2021). Histology of human teeth: Standard and specific staining methods revisited. Arch. Oral Biol..

[86] Galler K.M., Schweikl H., Thonemann B., D’Souza R.N., Schmalz G. (2006). Human pulp-derived cells immortalized with Simian Virus 40 T-antigen. Eur. J. Oral Sci..

[87] Dominici M., Le Blanc K., Mueller I., Slaper-Cortenbach I., Marini F.C., Krause D.S., Deans R.J., Keating A., Prockop D.J., Horwitz E.M. (2006). Minimal criteria for defining multipotent mesenchymal stromal cells. The International Society for Cellular Therapy position statement. Cytotherapy.

[88] Schmittgen T.D., Livak K.J. (2008). Analyzing real-time PCR data by the comparative *C*_T_ method. Nat. Protoc..

